# Efficiency of Microorganisms and Effectiveness of Biodegradation Techniques on LDPE Plastics: A Systematic Review

**DOI:** 10.12688/f1000research.151338.1

**Published:** 2024-07-04

**Authors:** Jorge Guillermo Morales Ramos, Leydy Mekinley Fernández Tarrillo, Anghelly Xiomara Guevara Bravo, Marilin Sánchez-Purihuamán, Carmen Rosa Carreño Farfán, Carolina Susana Loayza Estrada, Enrique Guillermo Llontop Ynga, Horacio De La Cruz Silva

**Affiliations:** 1 Facultad de Ciencias de la Salud, Escuela de Medicina Humana, Universidad Señor de Sipán, Lambayeque - Perú, Chiclayo, Lambayeque, 14001, Peru; 2 Facultad de Ciencias de la Salud, Universidad Señor de Sipán, Lambayeque - Perú, Chiclayo, Lambayeque, 14001, Peru; 3 Facultad de Ciencias Biológicas, Universidad Nacional Pedro Ruiz Gallo, Lambayeque - Perú., Lambayeque, Lambayeque, 14000, Peru

**Keywords:** Low-density polyethylene; efficiency; biodegradation; microbial consortia; fungi; bacteria.

## Abstract

**Introduction:**

The aim of the research was to demonstrate the efficiency of microorganisms and the effectiveness of biodegradation techniques on Low-density polyethylene (LDPE) plastics. The research question was: What is the efficiency of *LDPE*-degrading microorganisms and the effectiveness of biodegradation techniques?

**Methods:**

The systematic review was based on the Preferred Reporting Items for Systematic Reviews and Meta-Analyses (PRISMA) statement. Articles were obtained from Scopus, Web of Science (WOS), Embase, and Google Scholar. The DeCS/Mesh search terms were: Low-density polyethylene, efficiency, biodegradation, microbial consortia, fungi, bacteria. Inclusion criteria were: scientific articles that included bacteria, fungi, and microbial consortia reported as *LDPE* degraders that report the percentage of weight loss; articles published from January 2010 to October 2022, and publications in Spanish and English with open access. Exclusion criteria were: studies that do not report gravimetry, the biodegradation time of *LDPE*, and the genus or species of the polyethylene-degrading microorganism.

**Results:**

Out of 483 studies found, 50 were included in this Systematic Review (SR). The most frequent study techniques were scanning electron microscopy (SEM), gravimetry, and fourier transform infrared spectroscopy (FTIR), and in the case of microorganisms, the most studied belonged to the genus Pseudomonas, Bacillus, and Aspergillus. Regarding the isolation place, the most frequent mentioned in the reviewed articles were landfill soil and sanitary landfill soil. The efficiency of *LDPE*-degrading microorganisms was higher in bacteria such as *Enterobacter spp.*, *Pantoea spp.*, *Pseudomonas spp.*, *Escherichia coli*, and *Bacillus spp.*, which obtained a range of DE of 9.00-70.00%, 24.00-64%, 1.15 – 61.00%, 45.00%, and 1.5-40% with DT of 4-150, 120, 4-150, 30, and 30-120 days, respectively; in the case of fungi, the main microorganisms are *Neopestalotiopsis phangngaensis*, *Colletotrichum fructicola*, and *Thyrostroma jaczewskii* with efficiencies of 54.34, 48.78, and 46.34%, in 90 days, respectively; and the most efficient microbial consortia were from *Enterobacter spp.* and *Pantoea sp.* with 38.00 – 81.00%, in 120 days; and, *Pseudomonas protegens*, *Stenotrophomonas sp.*, *B. vallismortis* and *Paenibacillus sp.* with 55. 00 – 75.00% in 120 days.

**Conclusions:**

The most efficient microorganisms in *LDPE* degradation are *Enterobacter spp.*, *Pantoea spp.*, *Pseudomonas spp.*, *Escherichia coli*, and *Bacillus spp.*; in fungi *Neopestalotiopsis phangngaensis*, *Colletotrichum fructicola*, and *Thyrostroma jaczewskii*; and in microbial consortia, those formed by *Enterobacter spp.* and *Pantoea sp.*, and that of *P. protegens*, *Stenotrophomonas sp.*, *B. vallismortis* and *Paenibacillus sp.*; and the most effective techniques used in *LDPE* biodegradation are SEM, gravimetry, and FTIR.

## Introduction

Plastics are synthetic polymeric molecules characterized by their versatility, lightness, low cost, and high durability. ^1^ Among the most common are polypropylene, polyethylene, nylon, and polycarbonate, which are considered highly persistent with a capacity for bioaccumulation; they also contaminate the soil, mainly cultivable areas, thus reducing the water filtration capacity and fertilization of plants. ^2^^,^^3^ Currently, they have become one of the most significant pollutants in marine ecosystems where most of these float and disintegrate into small fragments when exposed to the sun, taking the name of microplastics. ^4^

Plastics are classified into ^1^: easily degradable, which includes biologically phase plastics such as compostable and biodegradable ones, and ^2^ difficultly degradable, among which thermoset and thermoplastic plastics such as polypropylene (PP), polyvinyl chloride (PVC), polystyrene (PS), polytetrafluoroethylene (PTFE), and low-density polyethylene (LDPE) can be mentioned. ^5^ The latter, globally, are the most marketed, with a production of 390.7 million tons in 2021, of which 50% was produced in Asia and 22% in America. ^6^ Polyethylene is the most commonly used plastic in everyday life, accounting for 96% of all plastics on the market. ^7^

LDPE accounts for 64%, and is primarily used in the form of bags, wrappings, and containers, which are discarded after use. ^8^ The mismanagement of plastic waste increases daily, mainly in Asian countries such as China, Indonesia, the Philippines, Vietnam, Malaysia, Thailand, and in Western countries like the United States. Of the plastics produced, considered to be 18 billion metric tons, 6% are incinerated, 23% are reused, 62% are disposed of in landfills, and 9% are considered recycled. ^1^

It has been demonstrated in-vitro that the ingestion of plastics by living beings produces a high impact on fauna. It is mentioned that they cause neurotoxic and degenerative damages in rodents, marine invertebrates, fish, and mammals, who are exposed to the presence of high levels of microplastics. ^9^ Some studies in fish indicate that microplastic particles can cause oxidative damage to lipids in the gills and muscles, as well as neurotoxicity through the inhibition of acetylcholinesterase and alterations in neurotransmitter levels. ^10^

Humans, as an important component of the ecosystem, are also affected by plastic waste. It has been estimated that a weekly intake of microplastics (MPs) with values ranging between 0.1 and 5 g can be found bound to food and drinking water, thereby generating adverse health effects. ^11^ In the city of Beijing, China, an analysis of feces conducted on young people between 18 to 25 years old who consumed water and food revealed the presence of microplastics such as polypropylene with a size of 20 – 800 nm. ^12^ Another study in Mexico found up to 30 microplastic particles in a series of foods such as energy drinks, tea, sodas, and beers ^13^; another work conducted in Iran, in the analysis of bottled mineral water, found values of 8.5 ± 10.2 particles/L of PET, PS, PP. ^14^

MPs are a globally recognized problem due to their prevalence in natural environments and the food chain, as well as their high impact on human health. Plastics directly affect living beings, either through ingestion or toxicity. It is noted that they could act as vehicles for invasive species and by adsorption on their surface of other synthetic chemical pollutants such as polychlorinated biphenyls (PCBs), polycyclic aromatic hydrocarbons (PAHs), or organochlorines, currently used by the chemical industry, thus potentiating or synergizing their toxic power due to components they contain such as plasticizers, heavy metal additives, etc. ^15^ Studies on microbiota have allowed the assessment of the effect of MPs, especially PETs, on microbiota, demonstrating that they would act at the colon level, decreasing the values of *Staphylococcus spp.*, *Bifidobacterium spp.*, and *Clostridium spp.* ^16^

An important aspect to consider is the degradation process of plastics, such as with LDPE, which can take up to 400 years to decompose. ^17^ The process involves various microorganisms, mainly bacteria and fungi, that utilize mechanisms of photooxidation, thermal degradation, ozonation, mechano-chemical and catalytic degradation, and also biodegradation. ^5^ The capability of hydroxylases, lipases, and laccases enzymes, secreted by LDPE-degrading microorganisms, which are responsible for breaking the polymer chain into low molecular weight fragments, must be mentioned. ^18^ Extracellular enzymes play a very important role in biodegradation through the depolymerization of LDPE to form intermediate products that can be used as a carbon source by microorganisms, ^19^ as they oxidize, reduce, hydrolyze, esterify, and cut the internal molecular structure of the polymer. ^20^

Microorganisms accelerate and increase the degradation process, making them an alternative to reduce the accumulation of petroplastics in the environment. ^21^ There are reports of bacteria ( *Pseudomonas spp. and Bacillus spp.*) and fungi ( *Aspergillus spp. and Fusarium spp.*) that can degrade this plastic under laboratory conditions. ^22^^,^^23^ The use of more efficient microorganisms in the degradation of LDPE will allow the proper selection of bacteria or fungi with greater action and degradative efficiency of plastic. ^24^ At the industrial level, it will involve the handling of various effective methods of detection and quantification, such as gravimetry, scanning electron microscopy (SEM), ^25^^–^^30^ Fourier transform infrared spectroscopy (FTIR), and gas chromatography coupled with mass spectrometry (GC-MS), which complement the study of the polymer’s natural degradation. ^31^

The purpose of this systematic review is to demonstrate the efficiency of LDPE-degrading microorganisms and the efficacy of the main biodegradation techniques on this type of plastics. The research question is: Which of the microorganisms is the most efficient and what techniques are the most effective in the biodegradation of LDPE-type plastics?

## Methods

The PRISMA (Preferred Reporting Items for Systematic Reviews and Meta-Analyses) methodology was used, which is established for systematic reviews and meta-analysis statements. ^32^ The information was extracted from articles obtained from various databases such as: Scopus, Web of Science (WOS), Embase, and Google Scholar. The identification, screening, and eligibility of scientific articles were organized through the Zotero Bibliographic Manager. ^33^ The protocol of the systematic review was registered in PROSPERO (International prospective register of systematic reviews) under the number CRD42024506168.

The search strategy in all databases consisted of managing Boolean operators (AND, OR, and NOT), keywords (biodegradation, low-density polyethylene), years of publication (2010 – 2022), type of document (original article), language (Spanish, English), and open access publications. The DeCS/Mesh search terms were: Low-Density Polyethylene, LDPE, efficiency, biodegradation, microbial consortia, fungi, and bacteria.

The auxiliary search strategy included:

✓ **Scopus**

((TITLE-ABS-KEY (BIODEGRADATION) AND TITLE-ABS-KEY (LOW DENSITY POLYETHYLENE)) AND (LIMIT-TO (LANGUAGE,“English”) OR LIMIT-TO (LANGUAGE,“Spanish”)) AND (LIMIT-TO (PUBYEAR,2010) OR LIMIT-TO (PUBYEAR,2011) OR LIMIT-TO (PUBYEAR,2012) OR LIMIT-TO (PUBYEAR,2013) OR LIMIT-TO (PUBYEAR,2014) OR LIMIT-TO (PUBYEAR,2015) OR LIMIT-TO (PUBYEAR,2016) OR LIMIT-TO (PUBYEAR,2017) OR LIMIT-TO (PUBYEAR,2018) OR LIMIT-TO (PUBYEAR,2019) OR LIMIT-TO (PUBYEAR,2020) OR LIMIT-TO (PUBYEAR,2021) ORLIMIT-TO (PUBYEAR,2022)) AND (LIMIT-TO (EXACTKEYWORD,“Article”)) AND (LIMIT-TO (DOCTYPE,“ar”)) AND (LIMIT-TO (SRCTYPE,“j”)))

✓ **Web of Science (WOS)**

(ALL=(biodegradation)) AND ALL=(low density polyethylene) and 2022 or 2021 or 2020 or 2019 or 2018 or 2017 or 2016 or 2015 or 2014 or 2013 or 2012 or 2011 (Publication Years) and 28 Article (Document Types) and Article (Document Types) and All Open Access (Open Access) and English (Languages)

✓ **Embase**

“biodegradation AND polyethylene AND low AND density AND [2010-2022]/py AND [article]/lim AND ([english]/lim OR [spanish]/lim”

✓ **Google Scholar**

The advanced search in this database included the exact phrase “LDPE biodegradation”; at least one of the following terms: Fungi, bacteria, or microbial consortia; terms mentioned in all scientific articles. It was also possible to delimit the years and languages of publication for each study.

### Selection criteria

**Inclusion criteria**

• Scientific articles that included bacteria, fungi, and microbial consortia reported as LDPE degraders.
• Scientific articles that report the percentage of weight loss after the process.
• Articles published from January 2010 to October 2022.
• Publications in Spanish and English with open access.

**Exclusion criteria**

• Studies that do not report gravimetry.
• LDPE biodegradation time.
• Genus or species of the polyethylene-degrading microorganism.

The coordination and development of the review activities were carried out through the Zoom video chat software. To include the studies, their relationship with the research question was verified, based fundamentally on the terms: Biodegradation, LDPE, bacteria, and fungi. Then, for the quality assessment of the Systematic Reviews, Meta-analysis and a scientific article evaluation scale were used to ensure strict compliance with the inclusion and exclusion criteria mentioned in previous paragraphs. The web application used throughout the process of identification, selection, eligibility, and inclusion was Zotero. To collect relevant data from each report, PRISMA 2020 was used. Empirical articles were evaluated using an analytical rubric designed according to the parameters founded on the SSAHS scale by López-López E, Tobón S, JuárezHernández LG) for the consideration of scientific articles. Systematic reviews were evaluated using the Quality Assessment of Systematic Reviews and Meta-Analyses, utilizing an observation guide (checklist style as indicated by the National Heart, Lung, and Blood Institute). ^34^ Data systematization tables were used considering year, author(s), title, techniques, and place of isolation. In this last item, the place of origin of the microorganism (isolation), the identified microorganism, and the study technique (TE) were included. The variables for which relevant information was sought were: degradation efficiency (DE) of LDPE degrading microorganisms, biodegradation of plastics, degradation time (DT), and percentage of weight loss.

## Results

**Figure 1.**
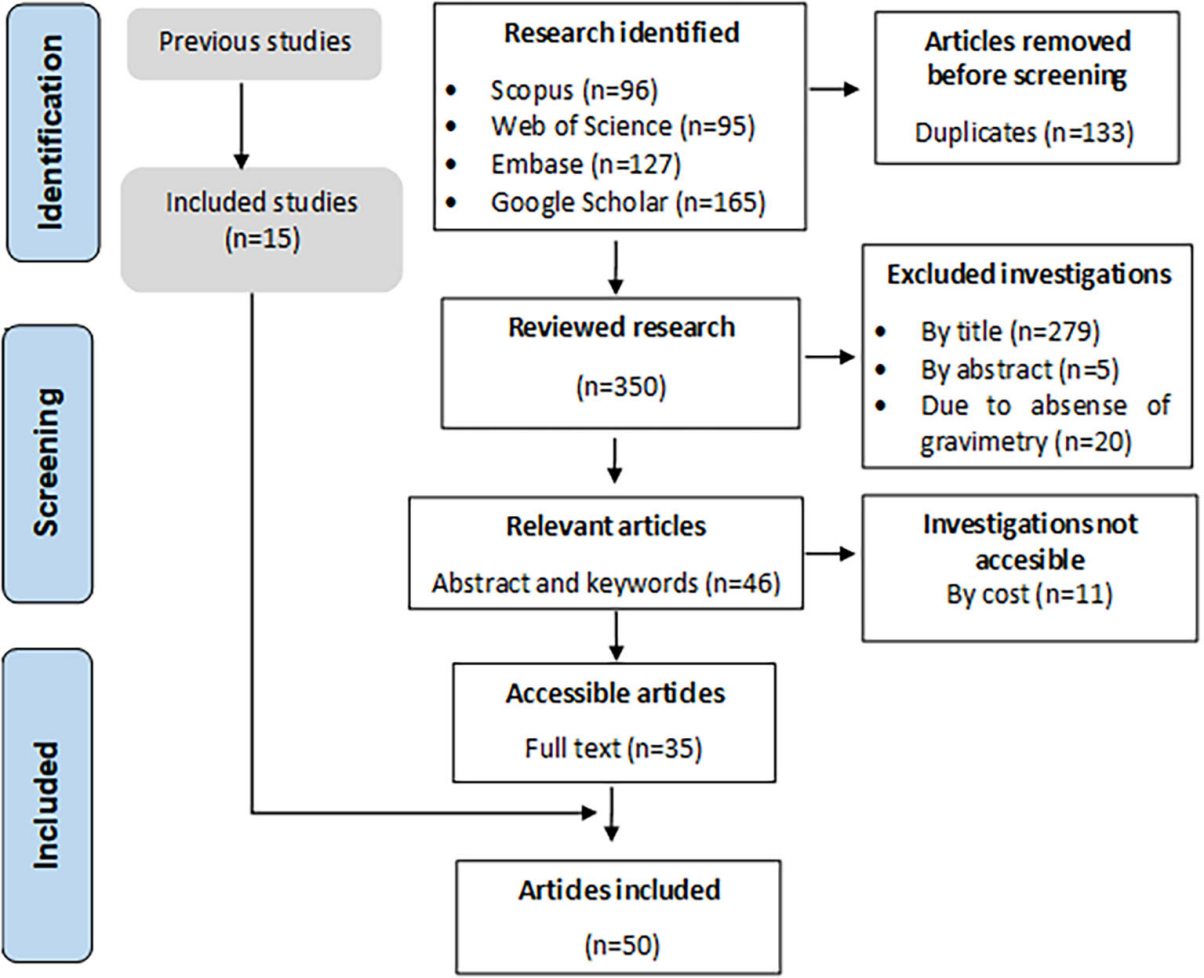
Flowchart of identification, screening, eligibility, and included articles.

Figure 1 shows the PRISMA methodology, whose search protocol identified 483 primary articles, of which 133 duplicates, 279 by title, 5 by abstract, 11 by access, and 20 for not meeting the inclusion terms were excluded. A total of 35 full-text articles were obtained, and 15 were included from previous review.

Table 1 indicates the number of articles found, totaling 50, with the highest quantity produced between the years 2018 to 2022. Also, it is observed that 100% of the articles correspond to experimental research works. Likewise, it points out the study techniques, with the most frequent being: SEM, gravimetry, and FTIR. Regarding the microorganisms identified in each of the studies, the most frequent in the phylum bacteria belonged to the genera *Pseudomonas* and *Bacillus*; as for the phylum of fungi, *Aspergillus spp.* predominated. As for the isolation site, the most used and mentioned in the reviewed articles were landfill soil and sanitary landfill. ^32^

**Table 1.** Articles Classified by Year, Author, Title, Study Type, Techniques, Isolation Location, and Identified Microorganisms.

N°	Year	Authors	Article title	Study type	Techniques and place of isolation and identified microorganisms
1	2010	Uribe D, Giraldo D, Gutiérrez S, Merino F. ^35^	Biodegradación de polietileno de baja densidad por acción de un consorcio microbiano aislado de un relleno sanitario, Lima, Perú.	Experimental	Isolation Location (IL): LDPE from landfill. Identified Microorganism (MI): *Pseudomonas sp.* MP3a., MP3b., *Penicillium sp.*, *Rhodotorula sp.*, *Hyalodendrun sp.* Study Technique (TE): Gravimetry, FTIR.
2	2012	Kyaw B, Champakalakshmi R, Sakharkar M, Lim C, Sakharkar K. ^28^	Biodegradation of Low Density Polythene (LDPE) by *Pseudomonas* Species.	Experimental	IL: Strain identified. MI: *P. aeruginosa* PAO1 (ATCC15729), *P. aeruginosa* (ATCC15692), *P. putida* (KT2440 ATCC47054), *P. syringae* (DC3000 ATCC10862). TE: Gravimetry, FTIR-ATR, SEM, GC-MS, Tensile strength.
3	2013	Tribedi P, Sil A. ^30^	Low-density polyethylene degradation by *Pseudomonas* sp. AKS2 biofilm.	Experimental	IL: Solid waste landfill soil. MI: *Pseudomonas sp.* AKS2. TE: Gravimetry, SEM, AFM, Tensile strength.
4	2014	Bhatia M, Girdhar A, Tiwari A, Nayarisseri A. ^25^	Implications of a novel *Pseudomonas* species on low density polyethylene biodegradation: an in vitro to in silico approach.	Experimental	IL: Sanitary landfill soil. MI: *P. citronellolis* EMBS027. TE: Gravimetry, FTIR, SEM, TGA
5	2014	Muthumani S, Anbuselvi S. ^36^	Waste management and enviornment protection – A study with isolation and characterisation of low density polyethylene degradating microbes.	Experimental	IL: Polyethylene trash. MI: *Streptococcus sp.*, *E. coli*, *Klebsiella sp.*, *Bacillus sp.*, *Pseudomonas sp.*, *Saccharomyces sp.*, *A. niger*, *A. flavus*, *Streptomyces sp.* TE: Gravimetry, Optical microscopy.
6	2014	Das M, Kumar S. ^26^	Microbial deterioration of low density polyethylene by *Aspergillus* and *Fusarium* sp.	Experimental	IL: Solid waste soil. MI: *Aspergillus sp.* (FSM-3, 5, 6, 8), *Fusarium sp.* (FSM-10). TE: Gravimetry, FTIR, SEM, Optical microscopy.
7	2015	Quinchía A, Maya S. ^29^	Degradabilidad de polietileno de baja densidad –LDPE- utilizando *Pycnoporus sanguineus* UTCH 03.	Experimental	IL: Strain identified. MI: *Pycnoporus sanguineus* UTCH03. TE: Gravimetry, FTIR, SEM, DSC.
8	2015	Duddu M, Tripura K, Guntuku G, Divya D. ^37^	Biodegradation of low density polyethylene (LDPE) by a new biosurfactant-producing thermophilic *Streptomyces coelicoflavus* NBRC 15399T.	Experimental	IL: Not mentioned. MI: *Streptomyces coelicoflavus* nbrc 15399. TE: Gravimetry, FTIR, Optical microscopy, BATH.
9	2015	Das M, Kumar S. ^38^	An approach to low-density polyethylene biodegradation by *Bacillus amyloliquefaciens.*	Experimental	IL: Strain identified. MI: *B. amyloliquefaciens* (BSM-1 and BSM-2). TE: Gravimetry, FTIR, SEM, Sturm.
10	2015	Deepika S, Jaya Madhuri R. ^39^	Biodegradation of low density polyethylene by microorganisms from garbage soil.	Experimental	IL: Garbage soil. MI: *Streptomyces sp*, *Pseudomonas sp*, *Aspergillus niger*, *A. flavus.* TE: Gravimetry.
11	2016	Gajendiran A, Krishnamoorthy S, Abraham J. ^27^	Microbial degradation of low-density polyethylene (LDPE) by *Aspergillus clavatus* strain JASK1 isolated from landfill soil.	Experimental	IL: Landfill soil. MI: *A. clavatus* JASK1. TE: Gravimetry, FTIR, SEM, Sturm, AFM.
12	2016	Abraham J, Gosh E, Mukherjee P, Gajendiran A. ^40^	Microbial Degradation of Low Density Polyethylene.	Experimental	IL: Sludge plus garden soil. MI: *Streptomyces sp.*, *A. nonius.* TE: Gravimetry, FTIR, GC-MS, Sturm, AFM.
13	2016	Skariyachan S, Manjunatha V, Sultana S, Jois C. ^41^	Novel bacterial consortia isolated from plastic garbage processing areas demonstrated enhanced degradation for low density polyethylene.	Experimental	IL: Plastic waste from rural and urban areas. MI: *Proteus spp.*, *Enterobacter spp.*, *Enterobacter spp.*, *Pseudomonas spp.*, *Pantoea spp.* TE: Gravimetry, GC-FID, SEM, Tensile strength, FTIR.
14	2017	Skariyachan S, Setlur A, Naik S, Naik A, Usharani M, Vasist K. ^42^	Enhanced biodegradation of low and high-density polyethylene by novel bacterial consortia formulated from plastic-contaminated cow dung under thermophilic conditions.	Experimental	IL: Landfill soil. MI: *P. protegens* bt-dsce02, *Stenotrophomonas sp.* bt-dsce03, *B. vallismortis* bt-dsce01, *Paenibacillus sp.* bt-dsce04. TE: Gravimetry, FTIR, SEM, EDS, NMR.
15	2017	Gajendiran A, Subramani S, Abraham J. ^43^	Effect of *Aspergillus versicolor* strain JASS1 on low density polyethylene degradation.	Experimental	IL: Landfill soil. MI: *A. versicolor.* TE: Gravimetry, FTIR, SEM, Sturm, AFM.
16	2017	Ojha N, Pradhan N, Singh S, Barla A., Shrivastava A, Khatua P, Rai V, Bose S. ^44^	Evaluation of HDPE and LDPE degradation by fungus, implemented by statistical optimization.	Experimental	IL: Landfill soil. MI: *P. oxalicum* NS4 (KU559906), *P. chrysogenum* NS10 (KU559907). TE: Gravimetry, FTIR, FE-SEM, AFM.
17	2017	Awasthi S, Srivastava N, Singh T, Tiwary D, Kumar P. ^45^	Biodegradation of thermally treated low density polyethylene by fungus *Rhizopus oryzae* NS 5.	Experimental	IL: Not mentioned. MI: *Rhizopus oryzae.* TE: Gravimetry, SEM, AFM, Tensile strength.
18	2018	Denisse Yans Z. Dela Torre, Lee A. Delos Santos, Mari Louise C. Reyes and Ronan Q. Baculi. ^46^	Biodegradation of low-density polyethylene by bacteria isolated from serpentinization-driven alkaline spring	Experimental	IL: Water from rock crevices of the Poon Bato spring in Botolan, Zambales, Philippines. MI: *Bacillus krulwichiae*, *Bacillus pseudofirmus*, *Prolinoborus fasciculus*, and *Bacillus sp.* TE: Gravimetry, SEM, FTIR.
19	2018	Munir E, Sipayung F, Priyani N, Suryanto D. ^47^	Potential of bacteria isolated from landfill soil in degrading low density polyethylene plastic.	Experimental	IL: Sanitary landfill soil. MI: *Streptococcus sp.* Sp2, *Streptobacillus sp.* Sp4. TE: Gravimetry, FTIR, SEM.
20	2018	Munir E, Harefa R, Priyani N, Suryanto D. ^48^	Plastic degrading fungi Trichoderma viride and *Aspergillus nomius* isolated from local landfill soil in Medan.	Experimental	IL: Landfill soil. MI: *Trichoderma viride*, *Aspergillus nonius.* TE: Gravimetry, Tensile strength.
21	2018	Hikmah M, Setyaningsih R, Pangastuti A. ^49^	The potential of Lignolytic *Trichoderma* isolates in LDPE (Low Density Polyethylene) plastic biodegradation.	Experimental	IL: Strain identified. MI: *Trichoderma spp.* (TL1), *Trichoderma spp.* (TL2), *Trichoderma spp.* (TL3). TE: Gravimetry, SEM, DSC, BATH.
22	2018	Thamizhmarai T, Kannahi M. ^50^	Biodegradation of low density polyethylene by *Aspergillus* sp. and Pseudomonas sp. isolated from plastic dumped site ─ A SEM analysis.	Experimental	IL: Vedharaniyam waste soil. MI: *Pseudomonas sp.*, *A. niger*, *A. flavus*, *A. oryzae.* TE: Gravimetry, FTIR, SEM.
23	2018	P. Priyadarshini, Summera Rafiq, SK. Jasmine Shahina, K. Vijaya Ramesh. ^51^	Biodegradation of Low Density Polyethylene (LDPE) by *Nocardiopsis alba* from municipal landfill in Chennai	Experimental	IL: In the area of the municipal solid waste landfill, Pallikaranai, Chennai, India. MI: *Nocardiopsis alba* TE: Gravimetry, FTIR, SEM.
24	2018	Jayaprakash V, Palempalli U. ^52^	Effect of Palmitic Acid in the Acceleration of Polyethylene Biodegradation by *Aspergillus oryzae.*	Experimental	IL: PE bags in soil for 6 months. MI: *A. oryzae.* TE: Gravimetry, FTIR, SEM.
25	2019	Sáenz M, Borodulina T, Diaz L, Banchon C. ^53^	Minimal Conditions to Degrade Low Density Polyethylene by *Aspergillus terreus* and *niger.*	Experimental	IL: Mangrove (Santay Island Ecuador). MI: *A. terreus*, *A. niger.* *TE*: Gravimetry, SEM.
26	2019	Bardají D, Furlan, J, Stehling E. ^54^	Isolation of a polyethylene degrading *Paenibacillus* sp. from a landfill in Brazil.	Experimental	IL: Solid waste landfill and incinerator. *MI*: *Paenibacillus sp.* TE: Gravimetry, FTIR, SEM.
27	2019	Islami A, Tazkiaturrizki T, Rinanti A. ^55^	The effect of pH-temperature on plastic allowance for Low- Density Polyethylene (LDPE) by *Thiobacillus* sp. and *Clostridium* sp.	Experimental	IL: Strain identified. MI: *Thiobacillus sp.* K29:AA29p, *Clostridium sp.* TE: Gravimetry, FTIR
28	2019	De Silva J, Jayasekera G, Nanayakkara C. ^56^	Identification of potential fungal degraders of low density polyethylene (LDPE).	Experimental	IL: Landfill soil. MI: _Fusarium sp._PS3, *Penicillium sp.* Ps2, *Aspergillus niger.* TE: Gravimetry, FTIR, SEM, Optical microscopy.
29	2019	Kartikey Kumar, Deepa Devi ^57^	Biodegradation of Low Density Polyethylene by Selected *Bacillus* sp.	Experimental	IL: Soil adhered to plastic. MI: *Bacillus sp.* ISJ51, ISJ55, and ISJ57. TE: Gravimetry, FTIR, SEM, BATH.
30	2020	Montazer Z, Najafi M, Levin D. ^58^	In vitro degradation of low-density polyethylene by new bacteria from larvae of the greater wax moth, *Galleria mellonella.*	Experimental	IL: Larvae. MI: *Cupriavidus necator* H16, *P. putida* LS46, *P. putida* IRN22, *Lysinibacillus fusiformis*, *Bacillus aryabhattai.* TE: Gravimetry, GC-FID.
31	2020	Butrón S. ^59^	Capacidad de biodegradación de *Pseudomonas aeruginosa* frente al polietileno de baja densidad.	Experimental	IL: LDPE from dumpsite. MI: *P. aeruginosa.* TE: Gravimetry, optical and fluorescence microscopy.
32	2020	Gupta K, Devi D. ^20^	Characteristics investigation on biofilm formation and biodegradation activities of *Pseudomonas aeruginosa* strain ISJ14 colonizing low density polyethylene (LDPE) surface.	Experimental	IL: Strain identified. MI: *P. aeruginosa* ISJ14. TE: Gravimetry, FTIR, FE-SEM, BATH.
33	2020	Dey A, Bose H, Mohapatra B, Sar P. ^60^	Biodegradation of Unpretreated Low-Density Polyethylene (LDPE) by *Stenotrophomonas* sp. and *Achromobacter* sp., Isolated From Waste Dumpsite and Drilling Fluid.	Experimental	IL: Plastic landfill waste. MI: *Stenotrophomonas sp.* P2, *Achromobacter sp.* DF22. TE: Gravimetry, FTIR, SEM, AFM, BATH.
34	2020	Sarker R, Chakraborty P, Paul P, Chatterjee A, Tribedi P. ^61^	Degradation of lowdensity poly ethylene (LDPE) by *Enterobacter cloacae* AKS7: a potential step towards sustainable environmental remediation.	Experimental	IL: Agricultural soil (West Bengal). MI: *Enterobacter cloacae* AKS7. TE: Gravimetry, SEM, BATH, Tensile strength, Fluorescence microscopy.
35	2020	Samanta S, Datta D, Halder G. ^62^	Biodegradation efficacy of soil inherent novel sp. *Bacillus tropicus* (MK318648) onto low density polyethylene matrix	Experimental	IL: Downtown Durgapur landfill, India. MI: *Bacillus tropicus* MK318648. TE: Gravimetry, FTIR, SEM, AFM, Tensile strength.
36	2021	Glen Cletus DSouza, Ryna Shireen Sheriff, Varun Ullanat, Aniruddh Shrikrishna, Anupama V. Joshi, Lingayya Hiremath, Keshamma Entoori ^63^	Fungal biodegradation of low-density polyethylene using consortium of *Aspergillus* species under controlled conditions	Experimental	IL: CI. MI: *Aspergillus niger*, *Aspergillus flavus* and *Aspergillus oryzae.* TE: Gravimetry, FT-IR and SEM.
37	2021	Maroof L, Khan I, Yoo H, Kim S, Park H, Ahmad B, Azam S. ^19^	Identification and characterization of low density polyethylenedegrading bacteria isolated from soils of waste disposal sites.	Experimental	IL: Landfill soil. MI: *B. siamensis*, *B. cereus*, *B. wiedmannii*, *B. subtilis*, *P. aeruginosa*, *Acinetobacter iwoffii.* TE: Gravimetry, FTIR, FE-SEM, XRD, Carbon analysis.
38	2021	Soleimani Z, Gharavi S, Soudi M, Moosavi Z. ^64^	A survey of intact low-density polyethylene film biodegradation by terrestrial Actinobacterial species.	Experimental	IL: Plastic landfill soil. MI: *Streptomyces* sp. IR-SGS-T10(MK719894.1), *Streptomyces* sp. IR-SGS-Y1(MK719896.1, *Streptomyces* sp. IR-SGS-T5(MK611552.1) *Streptomyces alborgiseolus* IR-SGS-T10(MK719894.1), *Streptomyces* sp. IR-SGS-K3(MK608706.1), *Streptomyces gancidicus* IR-SGS-K2(MH819728.1), *Streptomyces* sp. IR-SGS-K1(MK608363.1), *Rhodococcus ruber* IR-SGS-T6(MK611559.1), *Rhodococcus ruber* IR-SGS-T7(MK611560.1), *Nocardia* sp. IR-SGS-T9 (MK719893.1), *Nocardia farcinica* IR-SGS-T8 (MK719892.1). TE: Gravimetry, FTIR, SEM, Tensile strength.
39	2021	Waqas M, Haris M, Asim N, Islam H, Abdullah A, Khan A, Khattak H, Waqas M, Ali S. ^65^	Biodegradable Potential of *Bacillus amyloliquefaciens* and *Bacillus safensis* using low density polyethylene thermoplastic (LDPE) substrate.	Experimental	IL: Strain identified. MI: *B. safensis*, *B. amyloliquefaciens.* TE: Gravimetry, SEM.
40	2021	Khruengsai S, Sripahco T, Pripdeevech P. ^66^	Low-Density Polyethylene Film Biodegradation Potential by Fungal Species from Thailand.	Experimental	IL: Institute of Excellence in Fungal Research. MI: *D. italiana*, *T. jaczewskii*, *C. fructicola*, *S. citrulli*, *A. niger.* TE: Gravimetry, FTIR, SEM, GC-MS.
41	2021	Khandare S, Chaudhary D, Jha B. ^67^	Marine bacterial biodegradation of low-density polyethylene (LDPE) plastic	Experimental	IL: Strain identified. MI: *Cobetia sp.*, *Halomonas sp.*, *Exiguobacterium sp.*, *Alcanivorax sp.* TE: Gravimetry, FTIR-ATR, FE-SEM, AFM, TGA.
42	2021	Nadeem H, Alia K, Muneer F, Rasul I, Siddique M, Azeem F, Zubair M. ^68^	Isolation and identification of low-density polyethylene degrading novel bacterial strains	Experimental	IL: Solid waste landfills. MI: *Serratia sp.*, *Stenotrophomonas sp.*, *Pseudomonas sp.* TE: Gravimetry, FTIR.
43	2021	Chaudhary A, Chaitanya K, Dalmia R, Vijayakumar R. ^69^	Synergistic effect of UV, thermal, and chemical treatment on biological degradation of low-density polyethylene (LDPE) by *Thermomyces lanuginosus.*	Experimental	IL: Strain identified. MI: *Thermomyces lanuginosus.* TE: Gravimetry, FTIR, SEM, TGA.
44	2021	Skariyachan S, Taskeen N, Kishore A, Krishna B, Naidu G. ^70^	Novel consortia of *Enterobacter* and *Pseudomonas* formulated from cow dung exhibited enhanced biodegradation of polyethylene and polypropylene	Experimental	IL: Landfill soil. MI: *Enterobacter sp.* nov. bt DSCE01, *Enterobacter cloacae* nov. bt DSCE02, and *Pseudomonas aeruginosa* nov. bt. DSCE-CD03. TE: Gravimetry, FTIR, SEM, AFM, XRD, EDS.
45	2021	Zahari N, Abdullah S, Tuah P, and Cleophas F. ^71^	Biodegradation of low-density polyethylene (LDPE) and starch – based plastic (SBP) by thermophiles *Bacillus subtilis* and *Candida tropicalis*	Experimental	IL: Strain identified. MI: *B. subtilis*, *C. tropicalis.* TE: Gravimetry, FTIR, SEM.
46	2021	Perera P, Deraniyagala A, Mahawaththage M, Herath H, Rajapakse C, Wijesinghe P, Attanayake R. ^72^	Decaying Hardwood Associated Fungi Showing Signatures of Polyethylene Degradation	Experimental	IL: Dry reserve forest. MI: *Phlebiopsis flavidoalba*, *Schizophyllum commune*, *Phanerodontia chrysosporium.* TE: Gravimetry, FTIR, SEM, Tensile strength.
47	2022	Saira A, Maroof L, Iqbal M, Farman S, Lubna, Faisa S. ^73^	Biodegradation of Low Density Polyethylene (LDPE) Bags by Fungi Isolated from Waste Disposal Soil	Experimental	IL: Peshawar district landfills. *MI*: *Aspergillus Niger*, *Aspergillus flavus*, *Penicillium.* *TE*: Gravimetry, FTIR.
48	2022	Maleki M, Moghimi H, Azin E. ^74^	Biodegradation of thermo-oxidative pretreated lowdensity polyethylene (LDPE) and polyvinyl chloride (PVC) microplastics by *Achromobacter denitrificans* Ebl13.	Experimental	IL: Compost. *MI*: *Achromobacter denitrificans* Ebl13. *TE*: Gravimetry, Sturm, FTIR, SEM, TGA.
49	2022	Khruengsai S, Sripahco T, Pripdeevech P. ^75^	Microbial degradation of low-density polyethylene by *Neopestalotiopsis phangngaensis.*	Experimental	IL: Institute of Excellence in Fungal Research. *MI*: *Neopestalotiopsis phangngaensis.* *TE*: Gravimetry, SEM, Sturm, Tensile strength.
50	2022	Liu X, Zhang Y, Sun Q, Liu Z, Zhao Y, Fan A, Su H. ^76^	Rapid colonization and biodegradation of untreated commercial polyethylene wrap by a new strain of *Bacillus velezensis* C5.	Experimental	IL: Household waste landfill. *MI*: *Bacillus velezensis* C5. *TE*: Gravimetry, FTIR-ATR, SEM, EDS, FE-SEM, HTGPC, GC-MS.

Table 2 allows for the descriptive observation of microorganisms classified into bacteria, fungi, and microbial consortia, in quantities of 23, 17, and 9 respectively. Also, it specifies the species and the efficiency of LDPE-degrading microorganisms expressed in weight loss from highest to lowest and the days used for complete degradation, highlighting among bacteria *Enterobacter spp.*, *Pantoea spp.*, *P. spp.*, *Escherichia coli*, and *B. spp.* which obtained an ED range of 9.00-70.00%, 24.00-64.00%, 1.15 – 61.00%, 45.00%, and 1.50-40.00% with TD of 4-150, 120, 4-150, 30, and 30-120 days, respectively; in the case of fungi, the main microorganisms are *Neopestalotiopsis phangngaensis*, *Colletotrichum fructicola*, and *Thyrostroma jaczewskii* with ED of 54.34, 48.78, and 46.34%, respectively, and TD of 90 days; and, the most efficient microbial consortia were from *E. spp.* and *Pantoea sp.* with ED of 38.00 – 81.00%, and TD of 120 days; and, *P. protegens*, *Stenotrophomonas sp.*, *B. vallismortis*, *Paenibacillus sp.* with ED of 55.00 – 75.00% and TD of 120 days.

**Table 2.** Degradation efficiency (ED) of LDPE by microorganisms expressed as weight loss (%) and degradation time (TD) in days, 2010 – 2022.

Microorganisms		Degradation time (Days)	Degradation efficiency weight loss (%)
**Bacteria**			
	*Enterobacter spp.,* ^41^^,^^61^^,^^70^	45-120	9.00-70.00
	*Pantoea spp.* ^41^	120	24.00-64.00
	*Pseudomonas spp.* ^19^^,^^20^^,^^25^^,^^28^^,^^30^^,^^36^^,^^39^^,^^41^^,^^50^^,^^59^^,^^68^	4-150	*1.15*-61.00
	*Escherichia coli* ^36^	30	45.00
	*Bacillus* spp. ^19^^,^^36^^,^^38^^,^^47^^,^^57^^,^^58^^,^^62^^,^^65^^,^^71^^,^^76^	30-120	1.50-40.00
	*Proteus spp.* ^41^	120	16.00-59.00
	*Streptomyces spp.* ^36^^,^^37^^,^^39^^,^^40^^,^^64^	28-90	2.31-46.70
	*Serratia sp.* ^68^	150	40.00
	*Nocardiopsis alba* ^51^	150	32.25
	*Stenotrophomonas spp.* ^60^^,^^68^	100-150	7.54-32.00
	*Paenibacillus sp.* ^54^	90-120	11.60-30.80
	*Klebsiella sp.* ^36^	30	21.00
	*Achromobacter spp.* ^60^^,^^74^	60-100	7.45-12.30
	*Lysinibacillus fusiformis* ^58^	18	8.20
	*Rhodococcus spp.* ^64^	60	3.01-6.23
	*Nocardia spp.* ^64^	60	3.60-5.98
	*Prolinoborus fasciculus* ^47^	90	5.10
	*Halomonas sp.* H-255 ^68^	90	1.72
	*Cobetia sp.* H237 ^68^	90	1.40
	*Exiguobacterium sp.* H256 ^68^	90	1.26
	*Alcanivorax sp.* H265 ^68^	90	0.97
	*Acinetobacter iwoffii* ^19^	90	0.76
	*Streptococcus spp.* ^36^^,^^47^	30	0.16
**Fungi**			
	*Neopestalotiopsis phangngaensis* ^75^	90	54.34
	*Colletotrichum fructicola* ^66^	90	48.78
	*Thyrostroma jaczewskii* ^66^	90	46.34
	*Stagonosporopsis citrulli* ^66^	90	45.12
	*Diaporthe italiana* ^66^	90	43.90
	*Saccharomyces* ^36^	30	43.00
	*Aspergillus spp.* ^26^^,^^27^^,^^36^^,^^39^^,^^40^^,^^43^^,^^48^^,^^50^^,^^52^^,^^53^^,^^56^^,^^66^^,^^73^	30-270	4.90-40.60
	*Penicillium chrysogenum* NS10 *(KU559907)* ^73^	90	0.35-36.60
	*Schizophyllum commune* ^72^	60	9.65
	*Thermomyces lanuginosus* ^69^	30	9.21
	*Fusarium spp.* ^26^^,^^56^	60-90	0.59-9.00
	*Rhizopus oryzae* ^45^	30	8.40
	*Trichoderma spp.* ^48^^,^^49^	35-45	4.87-7.51
	*Candida tropicalis* ^71^	7	3.20
	*Phlebiopsis flavidoalba* ^72^	60	2.60
	*Phanerodontia chrysosporium* ^72^	60	2.50
	*Pycnoporus sanguineus* UTCH0 *3* ^29^	180	0.66
**Microbial Consortia**			
	*Enterobacter spp., Pantoea sp.* ^41^	120	38.00-81.00
	*P. protegens, Stenotrophomonas sp., B. vallismortis, Paenibacillus sp.* ^42^	120	55.00-75.00
	*Enterobacter sp.*nov. bt DSCE01 *, E.* *cloacae nov.* bt DSCE02, *P. aeruginpsa* nov. bt. DSCE-CD03 ^70^	160	64.25
	*Lysinibacillus xylanilyticus*, *A.* *niger* ^77^	126	15.80-29.50
	*A. niger, A. flavus, A. oryzae* ^63^	55	26.15
	*Cupriavidus necator* H16, ^58^ *P. putida* (LS46,IRN22) ^58^	18	13.50
	*Thiobacillus sp. K29, Clostridium sp.* ^55^	30	5.30-6.40
	*Pseudomonas spp.* (MP3a, MP3b) ^35^	60	5.40
	*Penicillium sp., Rhodotorula sp., Hyalodendron sp.* ^35^	60	4.80

LDPE: Low-density polyethylene. IL: Isolation Location. MI: Identified Microorganism *.* TE: Study Technique. FTIR: Fourier Transform Infrared Spectroscopy. FTIR-ATR: Fourier Transform Infrared Spectroscopy - Attenuated Total Reflectance. SEM: Scanning Electron Microscopy. GC-MS: Gas Chromatography–Mass Spectrometry. EDS: Energy Dispersive X-ray Spectroscopy. FE-SEM: Field Emission Scanning Electron Microscopy. HT-GPC: High Temperature Gel Permeation Chromatography. TGA: Thermogravimetric Analysis. AFM: Atomic Force Microscopy. XRD: X-ray Diffraction. BATH: Bacterial Adhesion to a Hydrocarbon. GC-FID: Gas Chromatography-Flame Ionization Detector. DSC: Differential Scanning Calorimetry. NMR: Nuclear Magnetic Resonance.

## Discussion

According to the data analyzed ( Table 1), in recent years there has been an increase in studies on LDPE-degrading microorganisms with the aim of minimizing environmental impacts through bioremediation. ^78^ The requirement for special technologies allows understanding the degree of polymer disintegration and the nature of its resulting products. In the SR, up to 20 study techniques used in the biodegradation of LDPE have been detected. From our point of view, we consider highlighting in this article those methods that, due to frequency and especially efficacy, stand out among others. These methods include the following: *SEM*, ^67^^,^^69^^–^^71^ *gravimetry*, ^25^^,^^26^^,^^27^^,^^28^^,^^29^^,^^30^^,^^35^^,^^36^^,^^39^^,^^40^ and *FTIR.* ^20^^,^^28^^,^^60^^,^^66^

The SEM study technique, ^19^^,^^42^^–^^44^^,^^46^^,^^49^^–^^54^^,^^56^^,^^62^^,^^63^^,^^70^^,^^72^ is used to detect the biodegradation of LDPE and is employed to monitor changes on the surface of the LDPE film. ^72^^,^^74^^–^^76^ The adhesion of microorganisms to the surface is essential for biodegradation. ^65^ After incubating LDPE with selected degrading microorganisms on the surface, some characteristics such as erosion, holes, and cavities are observed, which are attributed jointly to the formation of bacterial film and the penetration of fungal hyphae. ^60^^,^^68^ Erosion is considered the primary cause of the mass reduction of the surface due to the secretion of enzymes and microbial extracellular metabolites. ^20^

Another frequently used technique in the reviewed articles is gravimetry, ^20^^,^^42^^–^^46^^,^^48^^–^^55^^,^^68^^–^^71^ which is a simple and highly precise test to determine the polymer weight reduction, originating as a consequence of being used as a source of carbon and energy by microorganisms. ^19^^,^^62^^–^^65^^,^^72^^–^^74^^,^^76^ This weight loss is considered proportional to the surface area, as biodegradation starts on the polymer’s surface. ^38^ With Gram-positive bacteria, the degradation efficiency (DE) in terms of LDPE weight loss has been reported: *Streptomyces sp.* DE 5.2% and degradation time (DT) of 90 days ^40^ and *Bacillus amyloliquefaciens* DE 11% and DT 60 days. ^38^

It was found in the review that various articles mentioned the FTIR technique, ^19^^,^^25^^,^^26^^,^^27^^,^^28^^,^^29^^,^^30^^,^^35^^,^^37^^,^^40^^,^^44^^,^^46^^,^^50^^,^^51^^,^^64^^,^^67^^–^^70^^,^^74^^–^^76^ as the third most frequent of those applied to determine biodegradation; in addition, the cited studies consider it analytical and efficient, useful for identifying the chemical configuration of organic, polymeric, and inorganic material, and the morphological changes, which are supported by the chemical structural changes at the level of the carbon chains, observing new functional groups (alkoxy, acyl, carboxyls, and nitro) or absence of them, and modifications in the chains such as breaks, stretches, and formation of double bonds; moreover, this technique determines the carbonyl index (CI), which measures the degree of degradation of the LDPE and in which its value depends on the degraded carbonyl bonds. ^28^ In reality, it involves measuring the concentration of carbonyl groups (CG) corresponding to acids, aldehydes, and ketones. ^35^ In the process of LDPE biodegradation, the initial weight corresponds to the oxidation of the chain that leads to the formation of CG, and subsequently, these form carboxylic groups that are degraded by β-oxidation and then through the citric acid cycle to CO _2_ and H _2_O. ^77^

In Table 2, as observed, the microorganisms frequently reported in the articles analyzed in Table 1 include bacteria from the genera *Bacillus*, *Brevibacillus*, *Cellulosimicrobium*, *Comamonas*, *Delftia*, *Enterobacter*, *Escherichia*, *Idonella*, *Kocuria*, *Lysinibacillus*, *Paenibacillus*, *Pantoea*, *Pseudomonas*, *Rhodococcus*, *Rhodotorula*, *Stenotrophomonas*, and *Streptomyces*, ^25^^,^^28^^,^^30^^,^^42^^,^^46^^,^^58^^,^^59^ with the genera *Pseudomonas*, *Bacillus*, and *Streptomyces* predominating. Other microorganisms are fungi, with *Aspergillus sp.* most frequently cited. ^39^^,^^48^^,^^50^^,^^52^^,^^63^ Among the less frequently cited species include *Rhizopus oryzae*, *Paenibacillus sp.*, *Streptomyces coelicoflavus*, *Thiobacillus*, *Clostridium*, *Achromobacter denitrificans*, *Penicillium oxalicum*, *P. chrysogenum*, *Pycnoporus sanguineus*, *Enterobacter cloacae.* ^29^^,^^37^^,^^44^^,^^45^^,^^54^^,^^55^^,^^61^^,^^74^

In the same table, it is analyzed that among the most efficient bacteria in the degradation of LDPE according to the weight loss of the polymer include several species such as *Enterobacter spp.* with an ED of 9.00 – 70.00%, and TD of 4 -150, *Pantoea spp.* with an ED of 24.00 – 64.00% and TD of 120, *Pseudomonas spp.* with an ED of 1.15 – 61.00% and TD of 4-150, *Escherichia coli* with an ED of 45.00% and TD of 30, and finally, *Bacillus spp.* with an ED of 1.50 – 40.00% and TD of 30-120. *Bacillus sp.* is also considered as another important species in the biodegradation process, having a consumption rate of 0.0019 g of the polymer per day ^45^; or, participating in consortia such as the one constituted by *Bacillus vallismortis*, *Pseudomonas protegens*, *Stenotrophomonas sp.*, and *Paenibacillus sp.* ^42^

It has been determined that *P. aeruginosa* cultured on LDPE as the only carbon source has an ED of 0.0015 g of LDPE per day and a TD of 462 days to reduce a polyethylene film from 1g to 0.5g ^20^; *Enterobacter cloacae* AKS7 and *Escherichia coli* possess another type of degradative action, and it is due to the secretion of extracellular polymeric substances and the high hydrophobicity of the microorganism’s cell wall, which allows a greater formation and adherence of the bacterial biofilm. ^36^^,^^61^ In the case of *Pantoea sp.*, its efficiency can be measured either individually or in consortium with *Enterobacter.* ^41^

Fungi ( Table 2), like bacteria, are considered LDPE-degrading microorganisms. The most efficient are: *Neopestalotiopsis phangngaensis*, *Colletotrichum fructicola*, and *Thyrostroma jaczewskii* with EDs of 54.34, 48.78, and 46.34%, respectively, and a TD of 90 days. The mycotic activity is considered to be due to their great capacity for adherence. In the polymer biodegraded for 40 days, the biofilm formed by the strongly adhered fungi is observed; at 80 days, surface deformation is evident and microcracks are differentiated. ^28^ Other efficient species are also reported, such as *A. clavatus* with an ED of 35.00% and a TD of 90 days ^27^ and *A. versicolor* with 40.60% and a TD of 90 days. ^43^

The degradative efficiency of microbial consortia has also been reported in different studies, ^35^^,^^41^^,^^63^^,^^70^ showing the most efficient to be the one formed by *Enterobacter spp.* and *Pantoea sp.*, and the one of *Pseudomonas protegens*, *Stenotrophomonas sp.*, *B. vallismortis*, and *Paenibacillus sp.* The cooperation of different microorganisms allows the use of different and complementary metabolic capacities for their growth, forming pure or mixed biofilms (fungi and bacteria), more resistant and metabolically more active. ^79^

LDPE-degrading microorganisms form a biofilm on the polymer and use it as a carbon source for consumption, an event that is reflected in weight loss. The biodegradation by microorganisms is a process of high metabolic activity, in which the count of viable cells, the concentration of surface proteins, and the efficiency in degradation expressed as polymer weight loss must be taken into account. ^20^ The most frequent place of isolation of microorganisms with LDPE-degrading capacity came from landfill soil and sanitary landfills with the presence of plastics. Various studies have indicated that bacteria and fungi adapt under different environmental conditions, a process mediated by complex cellular changes at the enzymatic level, ^19^^,^^56^ maintain physiology and metabolism, thus ensuring the survival of microorganisms. It has been demonstrated that bacteria such as *Pseudomonas putida* is a resistant and efficient xenobiotic decomposer because it presents an effective efflux pump; similarly, *Streptomyces atacamensis* shows xerotolerant latency and spore response to desiccation, and upregulation of proteins that are functional during xeric stress, ^80^ which probably explains why certain microorganisms are more efficient at degrading LDPE compared to others.

Considering that degradation is a slow process (this activity occurs before 60 days of incubation), and that degradation methods are heterogeneous, some authors conclude that there is no standard methodology in relation to analytical methods. ^15^ However, in this article, we present the various biodegradation techniques, so a more precise vision could be had to assess which of them is the most consistent and effective according to their ED and TD.

Finally, based on the detection and quantification tests of polymer degradation, the exposed microorganisms constitute a sustainable alternative, useful for bioremediation and minimization of environmental impacts, with the aim of reducing environmental pollution by LDPE.

## Conclusions

• The microorganisms with the highest degradation efficiency on LPDE-type plastics in bacteria are *Enterobacter spp.*, *Pantoea spp.*, *Pseudomonas spp.*, *Escherichia coli*, and *Bacillus spp.*; in fungi *Neopestalotiopsis phangngaensis*, *Colletotrichum fructicola*, and *Thyrostroma jaczewskii*; and in microbial consortia, those formed by *Enterobacter spp.* and *Pantoea sp.*, and the one by *P. protegens*, *Stenotrophomonas sp.*, *B. vallismortis*, and *Paenibacillus sp.*
• The most effective techniques used in LDPE biodegradation are SEM, gravimetry, and FTIR.

### Limitations

The results obtained allow for the identification of a lack of studies on microorganisms efficient in the biodegradation of LDPE, which limits the possibility of expanding their number and understanding their efficiency. Moreover, there are few studies on alternative methods that are effective in biodegradation. These limitations should be taken into account for the guidance and development of new research.

#### Ethics and consent

Ethical approval and consent were not required.

## Data Availability

No data are associated with this article. DOI: https://doi.org/10.5281/zenodo.11447533
