# Supplementary material for: Efficiency of Microorganisms and Effectiveness of Biodegradation Techniques on LDPE Plastics: A Systematic Review
Source: F1000Res. 2024 Sep 2;13:745. Originally published 2024 Jul 4. [Version 2] doi: 10.12688/f1000research.151338.2 (PMC11289592; doi:10.12688/f1000research.151338.2)
Supplement: Peer review history

# Reviewer report: Reviewer response for version 2

**DOI:** 10.5256/f1000research.170129.r363815

**Published:** 2025-02-20

**Authors:** ASTRI RINANTI

**Affiliations:** 1 Environmental Engineering, Faculty of Landscape Architecture and Environmental Technology, Universitas Trisakti, Jakarta, DKI Jakarta, Indonesia

- The search method has been clearly explained, including the inclusion and exclusion criteria.
- Quantitative analysis of the degradation rate should be highlighted to strengthen the conclusion.
- The references used are numerous and relevant, but some should be updated with more recent studies from the past 3–5 years (2020–2024).
- The conclusion reflects the obtained results well but could be further strengthened with practical implications of the findings.
- It is recommended to add suggestions for future research on improving the efficiency of LDPE biodegradation.

Are the rationale for, and objectives of, the Systematic Review clearly stated?

Yes

Is the statistical analysis and its interpretation appropriate?

I cannot comment. A qualified statistician is required.

If this is a Living Systematic Review, is the ‘living’ method appropriate and is the search schedule clearly defined and justified? (‘Living Systematic Review’ or a variation of this term should be included in the title.)

Yes

Are sufficient details of the methods and analysis provided to allow replication by others?

Yes

Are the conclusions drawn adequately supported by the results presented in the review?

Yes

Reviewer Expertise:

Biotechnology Engineering, Environmental Engineering

I confirm that I have read this submission and believe that I have an appropriate level of expertise to confirm that it is of an acceptable scientific standard.

# Reviewer report: Reviewer response for version 2

**DOI:** 10.5256/f1000research.170129.r360173

**Published:** 2025-02-13

**Authors:** Laura Schaerer

**Affiliations:** 1 Colorado State University, Fort Collins, Colorado, USA

"Studies on microbiota have allowed the assessment of the effect of MPs, especially PETs, on microbiota, demonstrating that they would act at the colon level, decreasing the values of *Staphylococcus spp.*, *Bifidobacterium spp.*, and *Clostridium spp." - this sentence would benefit from additional clarification. "at the colon level" and "decreasing the values of" are not clear on what is being impacted or where.*

 "degradation process of plastics, such as with LDPE, which can take up to 400 years to decompose" - Many plastics take even longer. Biodegradation is highly variable in natural environments depending on conditions. Paper referenced is in Spanish, I am unable to confirm if this is a good reference. Perhaps cite additional papers and change this number to a range?

 Exclusion criteria - are these things that need to be in the papers, or cannot be in the papers. Unclear. 

 "The variables for which relevant information was sought were: degradation efficiency (DE) of LDPE degrading microorganisms, biodegradation of plastics, degradation time (DT), and percentage of weight loss." - does weight loss refer to loss of mass of plastic? could benefit from additional clarity.

 "However, in this article, we present the various biodegradation techniques, so a more precise vision could be had to assess which of them is the most consistent and effective according to their ED and TD." - this should be re-worded for clarity. Why is it difficult to determine which technique is most consistent and effective? 

 "The results obtained allow for the identification of a lack of studies on microorganisms efficient in the biodegradation of LDPE" - actually 50 studies is quite a lot! Why was a formal meta-analysis not done comparing effect sizes across articles?

 "The most effective techniques used in LDPE biodegradation are SEM, gravimetry, and FTIR." - these are not biodegradation techniques, these measure changes in the plastic due to biodegradation.

 Check spelling of "LDPE" in concluding paragraph.

 No formal meta analysis was performed - statistics would strengthen this review. Conclusions are largely observational reporting commonly observed organisms and most frequently used techniques.

Are the rationale for, and objectives of, the Systematic Review clearly stated?

Yes

Is the statistical analysis and its interpretation appropriate?

Not applicable

If this is a Living Systematic Review, is the ‘living’ method appropriate and is the search schedule clearly defined and justified? (‘Living Systematic Review’ or a variation of this term should be included in the title.)

Yes

Are sufficient details of the methods and analysis provided to allow replication by others?

Yes

Are the conclusions drawn adequately supported by the results presented in the review?

Yes

Reviewer Expertise:

Biodegradation, microbial metabolism, systems biology

I confirm that I have read this submission and believe that I have an appropriate level of expertise to confirm that it is of an acceptable scientific standard, however I have significant reservations, as outlined above.

# Reviewer report: Reviewer response for version 1

**DOI:** 10.5256/f1000research.165977.r300136

**Published:** 2024-07-30

**Authors:** Magaly de la Cruz Noriega

**Affiliations:** 1 Instituto de Investigación en Ciencias y Tecnología, University Cesar Vallejo, Lima District, Lima Region, Peru

This research work is very interesting and relevant, as it gives us updated information on the role of microorganisms in degrading low-density plastic. But for its indexing some details are required.

 In the introduction, it is necessary to include a more detailed paragraph on the plastic degradation techniques such as (thermal, radiation, mechanical, chemical and biological), then put emphasis on the biological ones, indicating the advantages and limitations in the biodegradation of low-density plastic, to have a clearer picture of this review, then eliminate the research question rather place the implication or impact that this work has.

 Regarding the results, consider in Table 1 to consider the classification as follows: (Year, Authors, Type of study, type of sample or sample, Technique (physical, mechanical or biological), identified microorganisms, Analysis in the detection of Degradation.

 The discussion needs to go deeper into Table 1.

Are the rationale for, and objectives of, the Systematic Review clearly stated?

Yes

Is the statistical analysis and its interpretation appropriate?

I cannot comment. A qualified statistician is required.

If this is a Living Systematic Review, is the ‘living’ method appropriate and is the search schedule clearly defined and justified? (‘Living Systematic Review’ or a variation of this term should be included in the title.)

Yes

Are sufficient details of the methods and analysis provided to allow replication by others?

Yes

Are the conclusions drawn adequately supported by the results presented in the review?

Partly

Reviewer Expertise:

biotechnology, bioremediation, electromicrobiology

I confirm that I have read this submission and believe that I have an appropriate level of expertise to confirm that it is of an acceptable scientific standard.

# Response

**Published:** 2024-08-06

**Authors:** Jorge Guillermo Morales Ramos

**Affiliations:** Lambayeque, Universidad De San Martin de Porres - Filial Norte, Chiclayo, Lambayeque, Peru

The observations made in terms of classifying degradation types and highlighting biodegradation among them were raised. The second point on table 1, the table was constructed on the basis of the suggestions, introducing the sample and techniques.

# Reviewer report: Reviewer response for version 1

**DOI:** 10.5256/f1000research.165977.r299819

**Published:** 2024-07-25

**Authors:** Claudio Quiñones-Cerna

**Affiliations:** 1 Escuela de Medicina, Facultad de Ciencias de la Salud, Universidad César Vallejo, Facultad de Ciencias Biológicas, Universidad Nacionalde Trujillo, Trujillo, Peru

The article titled "Efficiency of Microorganisms and Effectiveness of Biodegradation Techniques on LDPE Plastics: A Systematic Review" was published in F1000Research. This systematic review, based on the PRISMA method, examines studies published between January 2010 and October 2022, analyzing the degradation of LDPE by bacteria, fungi, and microbial consortia, as well as the techniques used to evaluate this degradation.

 The study is particularly relevant due to the growing concern about plastic accumulation in the environment and the negative impacts associated with these materials, such as soil and water contamination and the effects on human health and wildlife. The results of this study provide a comprehensive view of the biodegradative capabilities of different microorganisms and effective methodologies for evaluating plastic degradation, which can guide future research and industrial applications in plastic waste management.

 Main Comments

**Conceptualization of Biodegradation Techniques:**

*Comment:* It would be beneficial for the introduction of the study to include a detailed conceptualization of different biodegradation techniques, covering aerobic and anaerobic processes, necessary environmental conditions, types of microorganisms involved, and specific applications for LDPE plastics.

 This would help readers understand how these techniques are applied and evaluated in the context of plastic biodegradation, providing a clear and complete theoretical framework from the outset.

 Secondary Comments

**Diversity of Evaluation Techniques:**

*Comment:* The study highlights several evaluation techniques such as SEM, gravimetry, and FTIR but would benefit from a more in-depth discussion of the specific advantages and limitations of each technique.

*Rationale:* A detailed comparison would help readers choose the most suitable techniques for their own research and practical applications.

**Future Research in Microbial Consortia:**

*Comment:* The review mentions the effectiveness of microbial consortia in LDPE degradation but could be expanded to suggest additional studies exploring the synergistic interaction between different microorganisms.

 Understanding how microbial consortia interact and enhance the degradation of plastics can open new avenues for research and development of more efficient biotechnologies.

**Environmental and Human Health Impact:**

*Comment:* Although the impact of plastics on the environment and human health is addressed, a section specifically dedicated to discussing these effects with recent data and concrete examples would strengthen the study's argument.

 Providing detailed information on the environmental and public health consequences of plastic accumulation underscores the importance of developing and applying effective biodegradation techniques.

 The article offers a comprehensive and detailed overview of the biodegradative capabilities of various microorganisms and the effective methodologies for the degradation of LDPE. However, it could benefit from further conceptualization of biodegradation techniques and a more detailed explanation of the inclusion and exclusion criteria. Additionally, expanding the discussion on the advantages and limitations of evaluation techniques, synergy in microbial consortia, and the environmental and human health impacts would provide a more complete and applicable perspective.

Are the rationale for, and objectives of, the Systematic Review clearly stated?

Yes

Is the statistical analysis and its interpretation appropriate?

I cannot comment. A qualified statistician is required.

If this is a Living Systematic Review, is the ‘living’ method appropriate and is the search schedule clearly defined and justified? (‘Living Systematic Review’ or a variation of this term should be included in the title.)

Yes

Are sufficient details of the methods and analysis provided to allow replication by others?

Yes

Are the conclusions drawn adequately supported by the results presented in the review?

Yes

Reviewer Expertise:

Bioremediation and Biodegradation of organic contaminants

I confirm that I have read this submission and believe that I have an appropriate level of expertise to confirm that it is of an acceptable scientific standard.

# Response

**Published:** 2024-08-06

**Authors:** Jorge Guillermo Morales Ramos

**Affiliations:** Lambayeque, Universidad De San Martin de Porres - Filial Norte, Chiclayo, Lambayeque, Peru

We are grateful for this review of our article.
